# Supplementary material for: The Association between Circulating Branched Chain Amino Acids and the Temporal Risk of Developing Type 2 Diabetes Mellitus: A Systematic Review & Meta-Analysis
Source: Nutrients. 2022 Oct 20;14(20):4411. doi: 10.3390/nu14204411 (PMC9610746; doi:10.3390/nu14204411)
Supplement: Supplementary file 1 [file nutrients-14-04411-s001.zip › Supplementary S2 - PICOS.pdf]

Domain & Inclusion/Exclusion	Justification
**Patients:** *Inclusion*: Humans only - Adult participants (18+ years of age), irrespective of ethnicity, biological sex, or age; confirmed type 2 diabetes mellitus (T2DM) with a body mass index (BMI) of between 25-30.<br>*Exclusion:* Pregnant human participants, human participants under the age of 18, any non-human organism, patients with type 1 diabetes mellitus, patients with a BMI of less than 24 or more than 31.	Participants with T2DM and are overweight represent the population of interest.<br>We attempted to reduce the potential for confounding by controlling for BMI, diabetic status and sub group per study design.<br>We chose a BMI range of 25-30 as this is considered overweight range by the CDC and NHS
**Intervention:** Not applicable.	No intervention was assessed, as only prospective case-control studies are included.
**Comparison:**<br>*Inclusion*: With respect to the case group:<br>Inclusion: Overweight (BMI=25-30) population with laboratory-confirmed (either fasting glucose or glycated haemoglobin) T2DM.<br>Exclusion: The development of any significant co-morbidity across the group (e.g., cardiovascular, renal, and gastrointestinal), gestational diabetes, insulin-dependent type 2 diabetes mellitus.	Control groups must consist of BMI-matched individuals who otherwise match the baseline characteristics of the case group (those who develop type 2 diabetes mellitus), to limit confounding.<br><br>Development of co morbidities could potentially affect circulating serum BCAA levels as shown by [9].
**Observation:**<br>*Inclusion*: Serum branch chain amino acid (BCAA), either for the individual amino acids or the total count, reported as mmol/L, or an equivalent measurement that may be converted to mmol/L, to be reported as serum total count and reported as mmol/L. Any correlation data available will be used for qualitative analysis.<br>*Exclusion:* Any studies not reporting on an association between circulating BCAA levels and T2DM.	For this systematic review and meta-analysis, we only focused on the BCAAs and their metabolites. Since their discovery in the development of T2DM, the BCAAs are the most extensively researched set of amino acids [8]. In addition, BCAAs have consistently demonstrated an association between alterations in circulating BCAAs and T2DM [41], [42]. The associations between the remaining amino acids and T2DM and obesity are not as robust and not as widely researched as the BCAAs, they were the sole focus of this systematic review and meta-analysis [8].
**Study:**<br>Inclusion: Prospective case-control studies (only), with at least one full year of follow-up.<br>Exclusion: Randomised-control studies, cohort studies, review articles, meta-analyses, pre-print or non-peer reviewed materials, surveys, qualitative studies, a follow-up time of less than one year.	Follow up time of a year minimum was important to limit confounding.<br>Case-control studies were utilised in this systematic review for the following reasons:<br>1. Prior systematic reviews had used cohort trials - These yielded conflicting results, with some cohort studies demonstrating an inverse correlation between BCAAs with diabetes and obesity [22].<br>2. Further, case-control studies are preferred to cohort studies due to the inherent reduction in confounding.<br>3. The utilisation of cohort studies presupposes an exposure, which is independent of our research questions.<br><br>Due to our study considerations with respect to temporality, weight status, diabetic status and follow-up period, we address the majority of the recognised sources of confounding within the literature pertaining to this topic.<br>Our systematic review and meta-analysis is, therefore, a confounder-limiting reconsideration of the association between BCAAs with the overweight and diabetic disease state.
