# Supplementary material for: The Association between Circulating Branched Chain Amino Acids and the Temporal Risk of Developing Type 2 Diabetes Mellitus: A Systematic Review & Meta-Analysis
Source: Nutrients. 2022 Oct 20;14(20):4411. doi: 10.3390/nu14204411 (PMC9610746; doi:10.3390/nu14204411)
Supplement: Supplementary file 1 [file nutrients-14-04411-s001.zip › Supplementary S3 - PRISMA flow diagram.pdf]

# PRISMA 2009 Flow Diagram

**For:** "The Association between Circulating Branched Chain Amino Acids and the Temporal Risk of Developing Type 2 Diabetes Mellitus: A Systematic Review & Meta-Analysis"

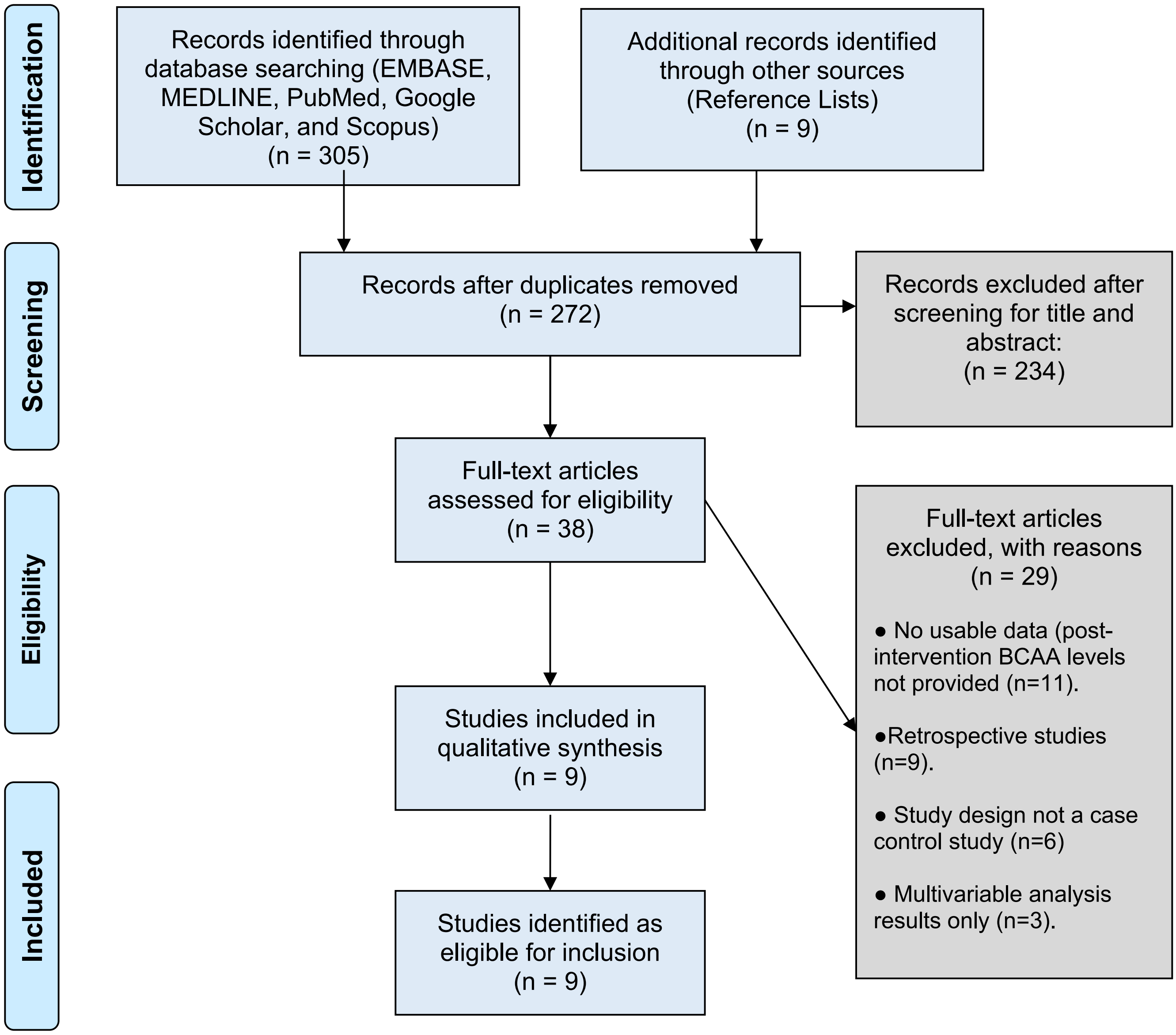


*From:* Moher D, Liberati A, Tetzlaff J, Altman DG, The PRISMA Group (2009). *P*referred *R*eporting *I*tems for *S*ystematic Reviews and *M*eta-*A*nalyses: The PRISMA Statement. PLoS Med 6(7): e1000097. doi:10.1371/journal.pmed1000097

**For more information, visit www.prisma-statement.org.**
