# Supplementary material for: The Association between Circulating Branched Chain Amino Acids and the Temporal Risk of Developing Type 2 Diabetes Mellitus: A Systematic Review & Meta-Analysis
Source: Nutrients. 2022 Oct 20;14(20):4411. doi: 10.3390/nu14204411 (PMC9610746; doi:10.3390/nu14204411)
Supplement: Supplementary file 1 [file nutrients-14-04411-s001.zip › Supplementary S4 - Risk of Bias.pdf]

	Selection					Comparability	Outcome			
STUDY TITLE	AUTHOR	Case Definition Adequate?	Representatives of the cases?	Selection of Controls	Definitio n of controls	Comparability of cases and controls on the basis of the design or analysis	Ascertainment of Exposure	Same method of ascertainment for cases and controls?	Non-response rate	Overall Score
Altered asparagine and glutamate homeostasis precede coronary artery disease and type 2 diabetes	F Ottosson [31]	★	-	★	★	★★	★	★	-	**7**
Metabolic signatures and risk of type 2 diabetes in a Chinese population: an untargeted metabolomics study using both LC-MS and GC-MS	A Stancáková [44]	★	-	★	★	★★	★	★	★	**8**
Plasma metabolites associated with type 2 diabetes in a Swedish population: a case–control study nested in a prospective cohort	L Shi [47]	★	★	★	★	★★	★	★	★	**9**
Metabolite profiles and the risk of developing diabetes (Framingham Heart Study)	T Wang [41]	★	-	★	★	★★	★	★	★	**8**
Metabolite Profiles and the Risk of Developing Diabetes (Malmö Heart Study)	T Wang [41]	★	-	★	★	★★	★	★	★	**8**
Identification of Serum Metabolites Associated With Risk of Type 2 Diabetes Using a Targeted Metabolomic Approach	A Floegel [42]	★	★	★	★	★★	★	★	-	**8**
Novel biomarkers for pre-diabetes identified by metabolomics	R Wang-Sattler [43]	★	★	★	★	★★	★	★	★	**9**
Hyperglycemia and a common variant of GCKR are associated with the levels of eight amino acids in 9,369 Finnish men	A Stancáková [44]	★	-	★	★	★★	★	★	-	**7**
Diabetes risk and amino acid profiles: cross-sectional and prospective analyses of ethnicity, amino acids and diabetes in a South Asian and European cohort from the SABRE (Southall And Brent REvisited) Study (South Asian Male Patients)	T Tillin [45]	★	-	★	★	★★	★	★	★	**8**
Diabetes risk and amino acid profiles: cross-sectional and prospective analyses of ethnicity, amino acids and diabetes in a South Asian and European cohort from the SABRE (Southall And Brent REvisited) Study (European Male Patients)	T Tillin [45]	★	-	★	★	★★	★	★	★	**8**
Metabolomic Profile Associated With Insulin Resistance and Conversion to Diabetes in the Insulin Resistance Atherosclerosis Study	ND Palmer [29]	★	★	★	★	★★	★	★	-	**8**
