# Supplementary material for: The Association between Circulating Branched Chain Amino Acids and the Temporal Risk of Developing Type 2 Diabetes Mellitus: A Systematic Review & Meta-Analysis
Source: Nutrients. 2022 Oct 20;14(20):4411. doi: 10.3390/nu14204411 (PMC9610746; doi:10.3390/nu14204411)
Supplement: Supplementary file 1 [file nutrients-14-04411-s001.zip › Supplementary S5 - All database search.pdf]

The full electronic search strategy performed for MEDLINE, EMBASE and PubMed are provided below:

Embase: (((obes* OR BMI OR overweight OR diabet* OR T2D*) AND (Branch* amino acid OR BCAA)) AND (prospective OR observation* OR case*)).ti,ab [English language] [Human age groups Adult 18 to 64 years OR Aged 65+ years] [Humans]

Medline: (((obes* OR BMI OR overweight OR diabet* OR T2D*) AND (Branch* amino acid OR BCAA)) AND (prospective OR observation* OR case*))

PubMed: (CASE REPORTS META-ANALYSIS MULTICENTRE STUDY OBSERVATIONAL STUDY REVIEW SYSTEMATIC REVIEW, HUMAN, ADULT: 19+ YEARS)

Google Scholar: (branched chain amino acid obesity diabetes observational study).

Scopus: TITLE-ABS-KEY ((((obes* OR bmi OR overweight OR diabet* OR t2d*) AND (branch* AND amino AND acid OR bcaa)) AND (prospective OR observation* OR case*))) AND (LIMIT-TO (PUBSTAGE , "final")) AND (LIMIT-TO (DOCTYPE , "ar") OR LIMIT-TO (DOCTYPE , "re")) AND (LIMIT-TO (SUBJAREA , "MEDI") OR LIMIT-TO (SUBJAREA , "BIOC")) AND (LIMIT-TO (EXACTKEYWORD , "Human")) AND (LIMIT-TO (LANGUAGE , "English")) AND (LIMIT-TO (EXACTKEYWORD , "Humans"))
